# Aphid reproductive investment in response to mortality risks

**DOI:** 10.1186/1471-2148-10-251

**Published:** 2010-08-17

**Authors:** Seth M Barribeau, Daniel Sok, Nicole M Gerardo

**Affiliations:** 1 Department of Biology, Emory University, 1510 Clifton Road, Atlanta GA, 30322, USA; 2 Institute of Integrative Biology (IBZ), ETH Zürich Universitätsstrasse 16, 8092 Zürich, Switzerland

## Abstract

**Background:**

Aphids are striking in their prodigious reproductive capacity and reliance on microbial endosymbionts, which provision their hosts with necessary amino acids and provide protection against parasites and heat stress. Perhaps as a result of this bacterial dependence, aphids have limited immune function that may leave them vulnerable to bacterial pathogens. An alternative, non-immunological response that may be available to infected aphids is to increase reproduction, thereby ameliorating fitness loss from infection. Such a response would reduce the need to mount a potentially energetically costly immune response, and would parallel that of other hosts that alter life-history traits when there is a risk of infection. Here we examined whether pea aphids (*Acyrthosiphon pisum*) respond to immunological challenges by increasing reproduction. As a comparison to the response to the internal cue of risk elicited by immunological challenge, we also exposed pea aphids to an external cue of risk - the aphid alarm pheromone (*E*)-*β*-farnesene (EBF), which is released in the presence of predators. For each challenge, we also examined whether the presence of symbionts modified the host response, as maintaining host fitness in the face of challenge would benefit both the host and its dependent bacteria.

**Results:**

We found that aphids stabbed abdominally with a sterile needle had reduced fecundity relative to control aphids but that aphids stabbed with a needle bearing heat-killed bacteria had reproduction intermediate, and statistically indistinguishable, to the aphids stabbed with a sterile needle and the controls. Aphids with different species of facultative symbiotic bacteria had different reproductive patterns overall, but symbionts in general did not alter aphid reproduction in response to bacterial exposure. However, in response to exposure to alarm pheromone, aphids with *Hamiltonella defensa *or *Serratia symbiotica *symbiotic infections increased reproduction but those without a facultative symbiont or with *Regiella insecticola *did not.

**Conclusions:**

Overall, our results suggest that pea aphids are able to increase their reproduction in response to specific cues and that symbiont presence sometimes moderates this response. Such increased reproduction in response to risk of death increases the fitness of both aphids and their vertically transmitted symbionts, and since these organisms have high reproductive capacity, slight increases in reproduction could lead to a very large numerical advantage later in the season. Thus both symbiotic partners can benefit by increasing host fecundity under dangerous conditions.

## Background

Hosts commonly respond to infection by mounting an immune response, but immunity is costly. Cytotoxic immune responses targeting pathogenic organisms can also damage host tissues [1,2], and activating the immune system reduces energy available for other purposes such as reproduction [3-5] and growth (reviewed in [6]). Because of these costs, mounting an immune response can reduce longevity [3,7]. Parasite resistance can also have tradeoffs even without immune activation. Parasite resistant hosts can have lower competitive ability [8], greater offspring mortality [9] and slower larval growth [10]. Given the costs of immunity, investment in immune capacity is intrinsically linked to aspects of host life history. For example, long-lived organisms optimize long term reproduction, forgoing rapid growth and reproduction, but must also survive to reproduce and are so 'required' to maintain a more effective, and presumably costly, immune system than short lived rapidly reproducing organisms [11,12].

Rather than mounting a costly immune response, hosts may change life-history traits to minimize the potential fitness loss once infected. Some hosts can increase their investment in reproduction in order to maintain fitness despite reduced longevity due to infection, or risk of infection, in a process termed fecundity compensation (a.k.a., terminal investment) [13-19]. The alternate strategies of fecundity compensation versus immune response can be considered as either investing in immediate benefits (increasing reproduction at a potential cost to survival if the infection induces mortality), or as an investment in long-term benefits (mounting an immune response that increases survival at a cost to immediate reproduction). However, neither strategy is foolproof. Investing in immediate reproduction when the subject is unlikely to die may result in costs to lifetime reproduction or may result in having offspring that may be poorly developed or born at a suboptimal time. In contrast, investing in an immune response will both reduce immediate reproduction and lifetime reproduction if the immune response fails to overcome the pathogen, or if the immune response itself is sufficiently damaging to the host. These two strategies are not entirely mutually exclusive but since both immunity and reproduction require considerable energetic investment we expect the contribution to one strategy to be dependent on commitment to the other. A number of other factors could also select for different or flexible reproductive schedules. Variable resource availability or cues of mortality risk, such as predation or intense competition, could select for plastic reproductive responses or alternative but static reproductive strategies. For example, risk of mortality from interspecific competitors has led to divergence in reproductive strategies in a guild of castrating trematode parasites [20]. Species that are likely to be outcompeted by conspecifics invest more in reproduction and less in growth than the dominant competitors [21].

Fecundity compensation has been described in a number of invertebrates. When the intensity of a parasitic mite infection is high, male *Drosophila nigrospiracula *increase their reproductive effort [18]. If *Biomphalaria glabrata *snails are infected with *Schistosoma mansoni *[16,17,19], or if the risk of infection is high [14], they increase their investment in early reproduction. Similarly, *Daphnia *water fleas [15] and *Acheta *crickets [13] increase reproductive effort in response to infection. Other cues of mortality could also be important in inducing fecundity compensation. For example, many insects release alarm pheromones when attacked that alert nearby conspecifics of danger (reviewed in [21]). These compounds could serve as indications of an increased risk of death and spur terminal investment in reproduction, especially when predators can be satiated, or when escape is unlikely.

Despite having numerous pathogenic and parasitic enemies, including fungi, bacteria, viruses, and parasitoid wasps, the immune system of pea aphids (*Acyrthosiphon pisum*) is reduced relative to other insects [22]. Pea aphids have, at best, weakly functioning lysozymes [22] and only one known antifungal peptide, thaumatin [23]. They lack a functional IMD pathway [22], which, in other insects, recognizes and responds to infection from Gram-negative bacteria, and some other microbes. Pea aphids also have an extremely limited encapsulation response [24-26], which defends against parasitoid wasp attack in other insects [27].

The reduced immune system of pea aphids may, in part, be tied to the close relationships aphids maintain with bacterial symbionts. Pea aphids can harbour several species of Gram-negative bacterial symbionts. All pea aphids have a 'primary', obligate symbiont, *Buchnera aphidicola*, which produces essential amino acids that are rare in the aphids' sugar-rich diet of phloem sap [28-31]. Pea aphids also frequently harbour one of three 'secondary', facultative symbionts, *Hamiltonella defensa *(a.k.a. T-type or PABS), *Regiella insecticola *(U-type, or PAUS), and *Serratia symbiotica *(R-type or PASS) [32]. Secondary symbionts are not required for survival or reproduction, but confer benefits under a variety of environmental conditions, including parasitoid wasp attack [26,33,34], fungal infection [33,35], and heat stress [36], and can alter host plant use [37-39]. The frequency of these facultative associations varies spatially [40] and temporally [41] but is maintained only at intermediate frequencies in wild populations, despite the benefits conferred [40]. These facultative symbionts are clearly ecologically important, and can influence fecundity under some conditions [42,43] but how they alter reproductive schedules is not well understood.

While aphids appear to have limited immune responses, they do have a well-characterized response to predation. When attacked by predators, aphids release the alarm pheromone (*E*)-*β *farnesene (EBF) that alerts nearby conspecifics of predator presence [44,45]. Exposure to this compound induces evasion behaviours such as dropping from the plant [46], but also increases the proportion of the exposed aphids' offspring that develop into the dispersing winged morph [47].

Pea aphids have the capacity for rapid reproduction, making fecundity compensation a potentially effective adaptive response to infection and other factors that increase the risk of death. Like other aphids, pea aphids are cyclically parthenogenetic, having a period of asexual reproduction during spring and summer and one generation of sexual reproduction in autumn. Asexually produced offspring are created apomictically and are genetically identical, barring mutation, to their mothers. When born, precocious clonal offspring already have developing embryos inside of them, facilitating a rapid generation time (i.e. telescoping generations). Because of this reproductive potential, a difference of a few rapidly reproducing clonal offspring could quickly multiply into a vast numerical difference in clone copies, and may facilitate establishment in new environments.

As part of a larger experiment on immune gene expression, Altincicek *et al. *[23] intriguingly demonstrated that when pea aphids of a single genotype were stabbed with a sterile needle or a needle contaminated with heat inactivated *Escherichia coli*, a commensal bacterium in pea aphids, the aphids increased their reproduction relative to unstabbed control aphids. Based on the work of Altincicek *et al*., it is unclear how general this response is across aphid genotypes, whether bacterial symbionts alter this response, or whether aphids respond to natural pathogens in the same fashion as *E. coli*. Aphid clones differ in reproduction [48] and thus may also differ in their reproductive responses to cues of mortality risk. Given the potential importance of increased reproduction to compensate for the aphids' relatively weak immune response, we expanded on Altincicek *et al*.'s work by challenging aphids with a natural pathogen. We examined fecundity compensation across multiple aphid lines, and with aphids that harbour secondary symbiotic bacteria. We also expanded the generality of our findings by exploring whether aphids respond to external cues of mortality risk, by exposing pea aphids to the alarm pheromone (*E*)-*β *farnesene (EBF), which signals the presence of predators in natural populations [44].

## Methods

### Subjects

We used four pea aphid clonal lines (5A, LSR1, G3, G6). These clones are maintained asexually on fava bean (*Vicia faba*) plants in 16 hr light: 8 hr dark conditions at 20°C. 5A and LSR1 were collected in Wisconsin in 1999 and New York in 1998, respectively, and G3 and G6 were collected in Georgia in 2008. G3, G6 and 5A clones did not originally harbour secondary bacterial symbionts. LSR1 was collected with *Regiella insecticola*. We used sublines of 5A with artificially established secondary symbionts (*Hamiltonella defensa*, *Regiella insecticola*, *Serratia symbiotica *or no secondary symbiotic bacteria) [34], and LSR1 both with and without *R. insecticola*. A subline of LSR1 was previously cleared of its secondary symbiont [49]. For each experiment, aphids were born within 24 hours of one-another to limit ontogenetic differences.

### Experiments

#### (1a) Do pea aphids increase reproduction when exposed to a bacterial pathogen? Does aphid genotype matter?

We challenged nine-day old aphids from four clonal lines that do not carry a secondary symbiotic bacteria (5A, LSR1, G3, G6) by either handling them as a control, stabbing them with a sterile minutin pin, or stabbing them with a minutin pin contaminated with a heat-killed bacterial pathogen. Twenty aphids from each line were exposed to every challenge. Heat-killed bacteria should serve as an immune elicitor without killing the host. We challenged aphids with the Gram-negative enteric (genus *Enterobacter*) bacterial pathogen, Ng5b. Ng5b was originally isolated from a laboratory pea aphid, and kills most experimentally infected aphids within 48 hours [22]. The day before the infection, we plated Ng5b from frozen glycerol stock onto Luria broth (LB) agar and incubated these plates at 37°C for 24 hours. On the morning of the infection, we transferred bacterial colonies to LB and incubated them at 37°C. We determined the concentration of broth cultures by optical density (OD600), and standardized the cultures to OD600 = 0.5. Aphids were stabbed dorsally in the abdomen as in [23]. We then allowed the aphids 30 minutes to heal in a clean Petri dish before we put them individually onto fava bean sprouts (approximately 10 days old) in a 16.5 × 14.9 cm zip-lock plastic bag with sufficient air to prevent the bag from compressing and crushing the aphid inside. We counted the number of offspring each aphid had every day and replaced the fava bean sprout every two days for six days. The number of offspring on day one (to measure the immediate response to challenge) and the cumulative fecundity from day two until day six (to measure the overall effect of the challenge independently of the analysis from day one) were analysed with analyses of variance (ANOVA) with aphid clone and exposure condition as factorial independent variables (JMP 8.0.1, SAS Institute Inc.). We chose day six to terminate monitoring reproduction as in pilot experiments we found that aphids challenged by stabbing with heat-killed bacteria returned to the control levels within this time (data not shown). Individuals that died before the experiment concluded were excluded from fecundity analyses. We used Tukey's HSD tests to determine the significant effects within treatments.

#### (1b) Do pea aphids increase reproduction when exposed to a bacterial pathogen? Does symbiont presence matter?

To determine whether the presence of secondary symbionts alters the reproductive responses of aphids to bacterial exposure we challenged nine-day old aphids from one clonal line (5A) carrying *Hamiltonella defensa*, *Regiella insecticola*, *Serratia symbiotica *or no secondary symbiotic bacteria as above. Twenty aphids from each symbiont condition were exposed to every challenge except those with *H. defensa *symbionts where seventeen individuals were exposed to each challenge. All other experimental and analysis methods were as above.

#### (2a) Do pea aphids increase reproduction in response to alarm pheromones? Does aphid genotype matter?

We exposed developing aphids to the alarm pheromone (*E*)-*β*-farnesene (EBF), or hexane as a control. Aphids were placed on fava bean plants and covered with transparent cups with mesh tops and then exposed to either EBF or a hexane control [47,50]. Twenty aphids of each of four clonal lines without secondary symbionts (5A, LSR1, G3, G6) were exposed to 5 *μ*L of 1000 ng/*μ*L EBF or to a 5 *μ*L hexane control (as in [47]) for a period of five days, starting when the aphids were one day old, by placing a 6 mm diameter paper disc, saturated with the exposure solution, on the top of a 200 *μ*L pipette tip and inserting the narrow end of the pipette tip into the soil of the plant pot. In previous experiments we found that this dose will reliably induce 100% winged offspring. Four days after the exposure period ended, when the aphids were nine days old, each aphid was then placed onto an individual fava bean sprout in boxes, and fecundity and survival was monitored for six days. We analyzed these data with ANOVA with the aphid clone and exposure condition as fixed factors.

#### (2b) Do pea aphids increase reproduction in response to alarm pheromones? Does symbiont presence matter?

We also explored how symbiont presence altered reproduction when exposed to alarm pheromones by exposing aphids from a clonal line (5A) with each symbiont, *H. defensa*, *R. insecticola*, *S. symbiotica *or no symbiont and a second line (LSR1) with *R. insecticola *or no symbiont Twenty aphids from each symbiont/line condition were exposed to every challenge. All experimental methods were as above. Aphid lines (5A and LSR1) were analysed separately with symbiont status and exposure condition as fixed factors in an ANOVA. We also analyzed whether aphids with the same symbiont condition from different lines responded to these stimuli in the same fashion with an additional ANOVA with aphid line, symbiont, and exposure as fixed effects.

#### (2c) Does symbiont presence alter behavioural responses to alarm pheromone exposure?

We tested the dropping behaviour of aphids exposed to EBF, hexane, or water to determine whether our exposure would influence the amount of time on plants and, in turn, alter reproduction; and whether symbiont presence alters this behaviour. We placed 10 fourth instar aphids from the 5A line with each symbiont condition (as in 2b) individually onto fava bean cuttings with approximately 50 mm long stalk and a single leaf in transparent polystyrene vials (30 mm diameter, 85 mm high, Thornton Plastics, Utah). We allowed the aphids to acclimate to their new environment for 20 minutes and then introduced a paper disc saturated with either EBF or hexane in the same concentration as 2a, or water. We then recorded whether or not aphids were on the plant and whether they were active (moving) every 30 s for 5 min. As the fourth instar is the last nymphal instar before maturity, we used these aphids because feeding activity immediately before maturity may have strong effects on final molt timing and subsequent reproduction. We analysed the proportion of time that each aphid was on the plant or active with a quasibinomial GLM in R (2.10.0, [51]) with symbiont and exposure as fixed effects.

## Results

### (1a) Do pea aphids increase reproduction when exposed to a bacterial pathogen? Does aphid genotype matter?

**Figure 1:**
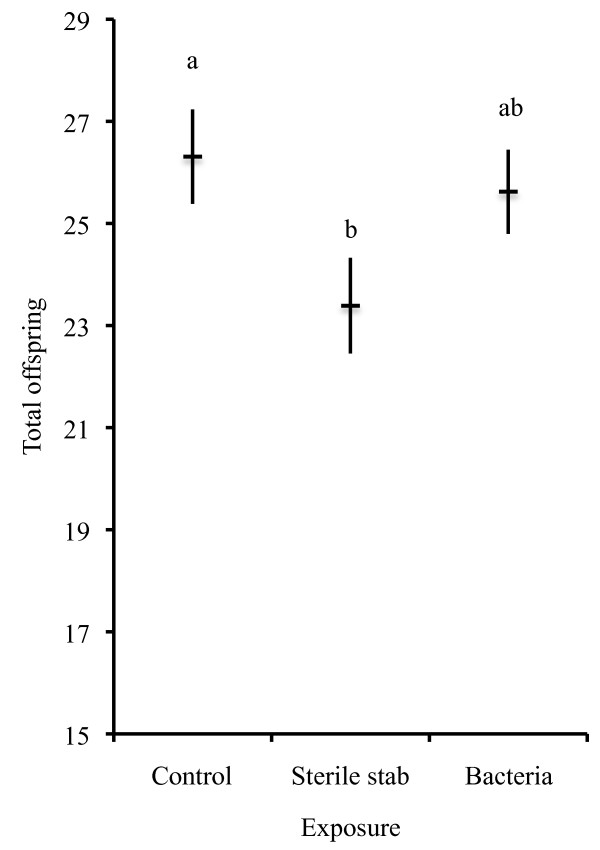
**Cumulative offspring between days 2 and 6 of aphids from multiple clonal lines, without facultative symbionts, when exposed to a bacterial challenge, sterile stab or handled as a control (experiment 1a)**. Cumulative offspring between days 2 and 6 $\left(\;\bar{X}\pm \text{ SE}\right)$ of aphids challenged with a bacterial stab, sterile stab, or handled as a control pooling four aphid lines without secondary symbionts. Groups with different letters are significantly different from one another based on Tukey's HSD test. Exposure did not alter reproduction immediately after challenge so is not presented.

Different clonal lines had different reproductive patterns with 5A and LSR1 aphids generally having higher reproduction ( day one and total days 2-6 mean ± SE: 5A = 8.70 ± 0.35, 28.50 ± 0.83; LSR1 = 8.53 ± 0.37, 27.16 ± 0.80) than both of our locally collected Georgia lines (G3 = 6.30 ± 0.42, 22.59 ± 1.21; G6 = 6.60 ± 0.43, 21.84 ± 1.02; Table 1a). On average, exposure did not significantly alter reproduction (Table 1a) although post-hoc tests indicate that control aphids had more offspring than aphids stabbed with a sterile needle (Figure 1 Tukey's HSD test, *P *= 0.045). Aphids stabbed with bacterially contaminated needles had greater fecundity than those stabbed with a sterile needle but were statistically indistinguishable from either the controls or those stabbed with a sterile needle (Figure 1, Tukey's HSD test vs. control *P *= 0.75, vs sterile stab *P *= 0.23). There was no significant interaction between aphid clone and exposure condition on reproduction (Table 1a).

**Table 1:** Statistical results from experiments 1a and 1b: fecundity in response to bacterial exposure.

	Day 1 offspring			Total offspring (Day 2-6)		
**1a: aphid line by bacterial exposure**	**d.f.**	***F***	***P***	**d.f.**	***F***	***P***

Line	3,228	10.15	< 0.0001	3,176	10.90	< 0.0001
Exposure	2,228	1.11	0.3300	2,176	3.00	0.0522
Line*Exp	6,228	0.78	0.5900	6,176	0.18	0.9819
						
	**Day 1 offspring**			**Total offspring (Day 2-6)**		

**1b: symbiont by bacterial exposure**	**d.f.**	***F***	***P***	**d.f.**	***F***	***P***

symbiont	3,219	9.94	< 0.0001	3,155	14.65	< 0.0001
Exposure	2,219	1.80	0.1700	2,155	7.84	0.0006
Symb*Exp	6,219	1.31	0.2500	6,155	1.15	0.3348

ANOVA statistics of fecundity data for experiments where aphids from different clonal lines (1a: 5A, G3, G6, LSR1) or with different secondary symbionts (1b: no symbiont, *Hamiltonella defensa*, *Regiella insecticola, Serratia symbiotica*) were challenged with heat-killed bacteria, a sterile stab, or were handled as a control.

### (1b) Do pea aphids increase reproduction when exposed to a bacterial pathogen? Does symbiont presence matter?

**Figure 2:**
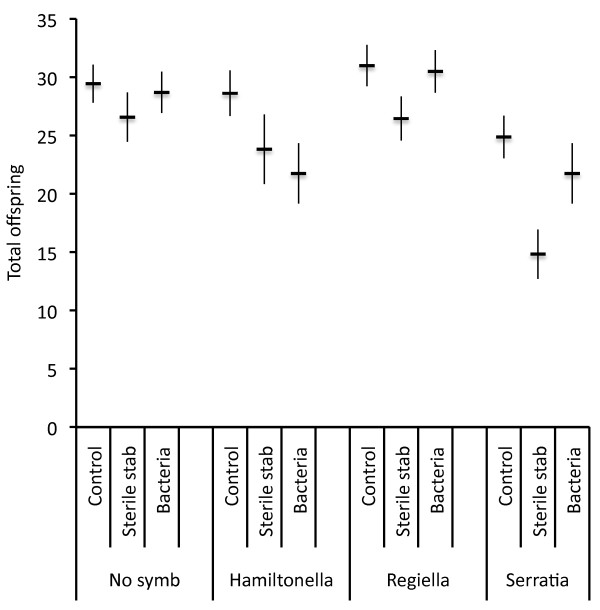
**Cumulative offspring between days 2 and 6 of aphids differing in symbiont status when exposed to a bacterial challenge, sterile stab or handled as a control (experiment 1b)**. Cumulative offspring between days 2 and 6 $\left(\;\bar{X}\pm \text{ SE}\right)$ for aphids from the 5A clonal line with *Hamiltonella defensa*, *Regiella insecticola, Serratia symbiotica *symbiotic infection or no secondary symbiont (No symbiont) challenged with a heat-killed bacteria or a sterile stab, or that were handled as a control. Fecundity differed significantly depending on the symbiont status, and the conditions to which aphids were exposed. Symbiont effects were similar immediately after challenge and exposure did not influence reproduction on the first day after challenge so these data are not shown.

Aphids with different symbionts had different reproductive output (Table 1b) with symbiont-free and *Regiella *hosting aphids having more offspring than aphids with *Serratia or Hamiltonella *(Figure 2). Exposure also significantly altered reproduction (Figure 2; Table 1b). Control aphids that were handled but not stabbed had higher reproduction than aphids that were stabbed with a sterile needle (total offspring Tukey's HSD *P = *0.0006) or bacterially contaminated needle (total offspring Tukey's HSD *P = *0.033). There was no significant interaction between symbiont status and exposure condition (Table 1b).

### (2a) Do pea aphids increase reproduction in response to alarm pheromones? Does aphid genotype matter?

The four clonal lines without secondary symbionts had different cumulative reproduction (day 2-6 mean ± SE: 5A = 42.66 ± 0.93, G3 = 38.15 ± 1.17, G6 = 39.50 ± 1.14, LSR1 = 45.00 ± 1.03; Table 2a) but did not have significantly different reproduction on day one (Table 2a). Exposure condition had no significant effect on reproduction (Table 2a); nor was there a significant interaction between aphid clone and exposure.

**Table 2:** Statistical results from experiments 2a-2c: fecundity and behavioural responses to alarm pheromone exposure.

	**Day 1 offspring**			**Total offspring (Day 2-6)**		
	
**2a: aphid line by EBF exposure**	**d.f.**	***F***	***P***	**d.f.**	***F***	***P***
	
Line	3, 189	0.40	0.7500	3, 171	7.81	< 0.0001
Exposure	1, 189	0.06	0.8000	1, 171	0.54	0.4600
Line*Exp	3, 189	1.13	0.3400	3, 171	0.77	0.5100
						
	**Day 1 offspring**			**Total offspring (Day 2-6)**		
	
***2bi: *symbiont by EBF exposure (5A)**	**d.f.**	***F***	***P***	**d.f.**	***F***	***P***
	
Symbiont	3, 147	10.49	< 0.0001	3, 127	1.80	0.1500
Exposure	1, 147	0.26	0.6100	1, 127	0.04	0.8400
Symb*Exp	3, 147	6.22	0.0005	3, 127	0.17	0.9200
						
	**Day 1 offspring**			**Total offspring (Day 2-6)**		
	
***2bii: *symbiont by EBF exposure (LSR1)**	**d.f.**	***F***	***P***	**d.f.**	***F***	***P***
	
Symbiont	1,76	11.03	0.0014	1,58	4.30	0.0430
Exposure	1,76	0.65	0.4220	1,58	0.72	0.4000
Symb*Exp	1,76	5.45	0.0220	1,58	2.86	0.0960
						
	**Day 1 offspring**			**Total offspring (Day 2-6)**		
	
**2b*iii*:line by symbiont by EBF exposure**	**d.f.**	***F***	***P***	**d.f.**	***F***	***P***
	
Line	1, 151	2.44	0.1202	1, 128	0.53	0.4699
Symbiont	1, 151	24.26	< 0.0001	1, 128	3.26	0.0732
Line*Symb	1, 151	0.36	0.5495	1, 128	2.83	0.0947
Exposure	1, 151	1.07	0.3015	1, 128	0.60	0.4417
Line*Exp	1, 151	4.36	0.0384	1, 128	0.43	0.5135
Symb*Exp	1, 151	0.005	0.7412	1, 128	0.94	0.3339
Line*Symb*Exp	1, 151	9.69	0.0022	1, 128	3.52	0.0629
						
	**Proportion of time on plant**			**Proportion of time active**		
	
**2c: symbiont by EBF behaviour (5A)**	**d.f.**	***χ^2^***	***P***	**d.f.**	***χ^2^***	***P***
	
Symbiont	3	11.38	0.7463	3	25.15	0.0007
Exposure	2	176.29	< 0.0001	2	97.38	< 0.0001
Symb*Exp	6	106.13	0.0753	6	6.90	0.5825

ANOVA statistics of fecundity data for experiments where aphids from different clonal lines (2a: 5A, G3, G6, LSR1) or with different symbionts (2b*i*: no symbiont, *Hamiltonella defensa*, *Regiella insecticola, Serratia symbiotica*; 2b*ii*: no symbiont, *Regiella insecticola*; 2b*iii*: 5A and LSR1 with no symbiont, or *Regiella insecticola*) exposed to the alarm pheromone EBF or hexane as a control. (2c) GLM statistics of the proportion of time aphids were either on their plant, or active after exposure to EBF, hexane or water.

### (2b) Do pea aphids increase reproduction in response to alarm pheromones? Does symbiont presence matter?

**Figure 3:**
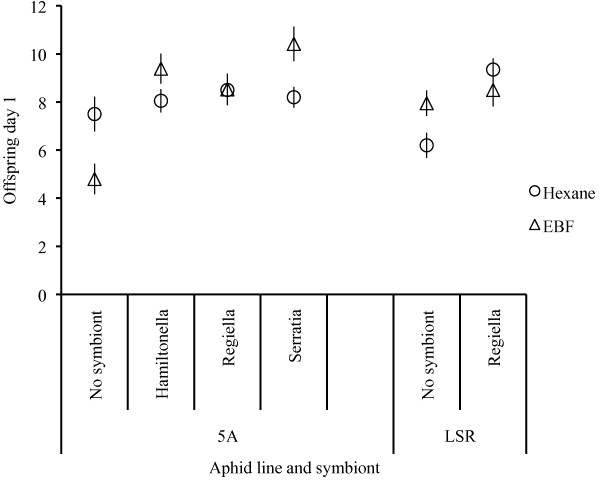
**Initial offspring of aphids differing in symbiont status when exposed to alarm pheromone or hexane (experiment 2b)**. Number of offspring $\left(\;\bar{X}\pm \text{ SE}\right)$ on day one for aphids (aphid line is given below symbiont status) with either *Hamiltonella defensa *(5A), *Regiella insecticola *(5A, LSR1), *Serratia symbiotica *(5A) symbiotic infection or with no secondary symbiont (5A, LSR1), challenged with EBF or hexane. A similar, but less pronounced pattern was apparent in cumulative reproduction.

Symbiont status significantly altered reproduction for both lines of aphids examined after one day (Figure 3. Table 2bi-ii), with aphids bearing symbionts having more offspring than those without symbionts. Aphids exposed to EBF or hexane had similar reproduction on average (Table 2bi-ii). Aphids with different symbionts, however, responded to the EBF challenge differently immediately after exposure (Figure 3; Table 2bi-ii). Aphids with *H. defensa *or *S. symbiotica *symbiotic infections increased their reproduction when exposed to EBF, whereas aphids that harboured *R. insecticola *had similar reproduction when exposed to EBF or the hexane control (Figure 3). Aphids from the different lines (5A and LSR) responded differently to exposure to EBF and symbiont status influenced this response differently in these lines (as indicated by line-by-exposure and line-by-exposure-by-symbiont interactions, Table 2biii). Aphids from the 5A line had fewer offspring when exposed to EBF than the hexane controls but in LSR aphids this pattern was reversed, whereas when either line carried *R. insecticola *their reproduction was similar regardless of exposure.

### (2c) Does symbiont presence alter behavioural responses to alarm pheromone exposure?

**Figure 4:**
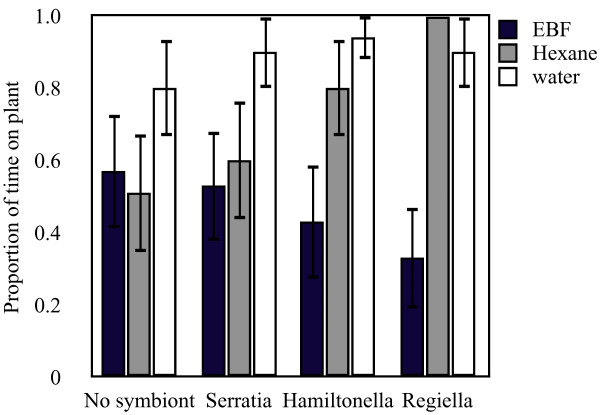
**Behaviour of aphids with different symbionts when exposed to alarm pheromone or hexane (experiment 2c)**. The proportion of time aphids (5A) with *Hamiltonella defensa*, *Regiella insecticola*, *Serratia symbiotica *symbiotic infection or no secondary symbiont spent on their plant after being exposed to the alarm pheromone EBF, hexane or water.

Aphids exposed to EBF were significantly less likely to be on their plant (47% of the time) and were more active (13% of the time) than either the hexane (73% of the time on plant, 1.5% of the time active) or water (89% of the time on plant, 0% of the time active) controls (Table 2c). Irrespective of exposure, the secondary symbiont status of the aphids did not significantly affect the amount of time spent on plants, but did affect the amount of time active (aphids with no secondary symbiont were active 9.3% of the time, with *R. insecticola *6% of the time, with *S. symbiotica *or *H. defensa *2% of the time; Table 2c). When exposed, however, aphids spent a different amount of time on their plants depending on which symbiont they harbour, although this interaction only approached significance (Figure 4; Table 2c) but did not alter the amount of time that they were active. Aphids with *R. insecticola *symbionts spent less time on their plants when exposed to the alarm pheromone than any other symbiont condition and aphids with *S. symbiotica *symbionts spent the least amount of time on plants when exposed to the hexane control (Figure 4).

## Discussion

Fecundity compensation may represent an effective response to risk of mortality especially in organisms like aphids with extremely limited immunity and explosive reproductive capacity. We found that some pea aphid clones increase their reproduction in response to risk cues. Exposure to alarm pheromone increased aphid reproduction in aphids carrying *Hamiltonella defensa *or *Serratia symbiotica *and in one secondary symbiont-free aphid clone, but decreased reproduction in the other symbiont-free clone. Aphids with *Regiella insecticola *symbionts had similar reproduction after exposure to EBF as controls. When stabbed with a sterile needle, aphids reduced their reproduction, indicating a general cost to wounding, but aphids without secondary symbionts that were exposed to heat killed bacteria had reproduction similar to control, unstabbed, levels, suggesting compensation for the stabbing by these aphids. This suggests that a number of factors, including the type of exposure and the presence of symbionts, must be taken into account when assessing the likelihood of a given aphid having a non-immunological response to stressors.

Previous work on a single aphid genotype suggested that exposure to *E. coli *drastically increases reproduction [23]. Our results differed from Altincicek *et al. *[23] in that aphids stabbed with a sterile needle reduced reproduction relative to controls, and while aphids given the bacterial stimulus increased their reproduction relative to the sterile stabbed aphids, we did not see the more than two-fold increase described in Altincicek *et al. *[23]. Although we based our methods on those used in Altincicek *et al*.'s paper, our methods did differ in some respects, which may explain the differences in our results (Altincicek, personal communication). First, our bacterial stimulus was an aphid pathogen as opposed to a commensal bacterium in Altincicek *et al. *[23]; exposure to an aphid pathogen, even when heat-killed, may also expose the aphids to bacterial toxins that are harmful, thereby reducing fecundity. Second, our wounding protocol used a smaller gauge needle and would have caused less damage as a result. Third, the environment that aphids were maintained in was more benign in our study than in Altincicek *et al. *[23]; we transferred aphids onto fava bean sprouts, which maintain plant turgor for several days, whereas the previous study kept aphids on leaves within Petri dishes. This environmental difference may also account for why we had considerably higher reproduction 24 hrs after challenge (6-10 offspring) relative to Altincicek *et al. *(0-3 offspring). Finally, there is considerable clone-level variation in reproduction, and although we did not detect any clone by exposure interaction in the set of clones examined here, other clones could potentially respond differently to these challenges. The differences between this study and the previous work are intriguing as the results suggest that when the exposure is more traumatic, and the environment poorer, as in Altincicek *et al. *[23], reproduction increases more dramatically than when the exposure and environment is more benign (this study). Together, these studies suggest that fecundity compensation exists in aphids, but that changes to aphid reproduction depend on the nature of the challenge and the subsequent risk to the aphids' survival.

Increasing reproduction in response to cues of risk of death, such as infection or predation risk would be advantageous when the cues reliably indicate risk. This benefit extends to all reproductively capable organisms but would be most important in species that are unable to respond to the risk at hand (e.g. aphids with their impoverished immune systems) because of the high cost of failing to reproduce when incapable of surviving the challenge. While the initial experiments examined how aphids respond to internal cues of mortality risk (i.e., cuticle damage and presence of bacterial antigens), we also found that external cues of risk alter reproductive responses. EBF exposure, an external cue of mortality risk, altered pea aphid reproduction differently for aphids with different secondary bacterial symbionts. Aphids with *Hamiltonella defensa *or *S. symbiotica *symbionts increased their reproduction when exposed to alarm pheromones relative to hexane treated controls. Both of these secondary symbionts also protect against heat shock [36,42]. If symbionts changed the behaviour of their hosts, perhaps by reducing their readiness to drop, they may feed more and thus have increased reproduction. The data from our behavioural assay, however, found that aphids with *S. symbiotica *symbionts, that increase reproduction when treated with EBF, spent as much time on their plant when exposed to EBF as the hexane control. In contrast, aphids with *R. insecticola *symbionts spent comparatively little time on their plants when exposed to EBF but had similar reproduction when exposed to hexane or EBF. It therefore seems unlikely that symbiont condition altered host behaviour sufficiently to change the exposed aphids' fecundity.

Our results, coupled with those of Altincicek *et al. *[23] suggest that aphids increase their reproduction in response to external (alarm pheromone), and internal (challenge with a heat-killed bacteria) cues of risk. We further found that this response to alarm pheromones depends both on the aphid clone and the symbiont they carry. This influence of symbiont on aphid fecundity when exposed to risk of death is intriguing as it raises questions about mechanisms of fecundity compensation in aphids. These facultative symbionts are primarily vertically transmitted; therefore, aphid reproduction is required for symbiont reproduction. In turn, aphid fecundity compensation in response to risk of death benefits both aphids and their symbionts.

## Conclusions

The limited ability of pea aphids to mount a strong immune response to bacterial pathogens could increase the importance of fecundity compensation in response to infection. Because of their prodigious reproductive capacity, small increases in aphid fecundity can quickly multiply and may drastically alter clone level competitive interactions. Our results suggest that pea aphids are able to increase their reproduction in response to specific cues and that symbiont presence sometimes moderates this response. Aphids, even with their extremely rapid reproduction, are, under some circumstances, reproducing at a rate below their capacity, which is perplexing. Identifying the presumed costs associated with early reproduction in aphids and determining how aphids respond to other ecologically relevant cues of increased risk of death, such as parasitic wasp attack, declining host-plant condition, or the presence of predators, remains to be explored. Future work should also determine how the presence of bacterial symbionts alters reproduction across these conditions, and, ultimately, how changes in reproductive effort alter clone-level competitive dynamics.

## Authors' contributions

SMB and NG designed the study. SMB and DS conducted the experiments. SMB analyzed the data. SMB, NG and DS wrote the manuscript. All authors read and approved the final manuscript.
